# Suggestions for a standard clinical practice curriculum and learning objectives for physical therapy education in Korea

**DOI:** 10.3352/jeehp.2017.14.23

**Published:** 2017-10-19

**Authors:** Tae Young Oh, Kyung Soon Lee, Byung Jo Kim

**Affiliations:** 1 Department of Physical Therapy, Health and Welfare College, Silla University, Busan, Korea; 2 Department of Physical Therapy, Dongju University, Busan, Korea; 3 Department of Physical Therapy, College of Nursing, Healthcare Sciences and Human Ecology, Dong-Eui University, Busan, Korea; Hallym University, Korea

Since 1965, the Korean physical therapist licensing examination has been conducted to foster professional physical therapists. As the responsibilities of a physical therapist include measuring and evaluating patients’ physical function and developing the competence to establish treatment plans and predict the outcomes thereof, the need for a new examination system to help develop these abilities has been pointed out. This idea led to the introduction of case-based items on the examination in 2014, and it is expected that case-based items will replace all 60 existing practice items [1]. It is necessary to formulate a standardized curriculum and learning objectives for clinical practice before developing clinical cases based on clinical practice and case-based items. The present study aimed to suggest a standard clinical practice curriculum and learning objectives for physical therapy education in Korea to facilitate the development of case-based items for the Korean physical therapist licensing examination.

We conducted a survey of the programs of 14 universities and 19 colleges using a questionnaire to collect information about various characteristics of clinical practice from June to September 2016. Raw data were available from Supplement 1. Ten researchers and 12 advisers established a standard clinical practice curriculum and learning objectives by analyzing the collected data using descriptive frequency analysis and discussions using the Developing a Curriculum method. Through this method, it was possible to create an educational program capable of reflecting the tasks and techniques of each job.

## Ethical approval

This research was authorized by the Institutional Review Board of Silla University (1041449-201708-HR-008).

## The current status of clinical practice in physical therapy education in Korea

Representatives from 19 colleges and 14 universities answered and returned the questionnaires. The results of the survey are shown in Table 1.

## Establishment of a standard clinical practice curriculum

A curriculum was designed that prescribed a period of 16 weeks of clinical practice, including 4 weeks of in-school training. Therefore, there were 640 hours of practice with 40 hours of practical training as suggested by the Korean Accreditation Board of Physical Therapy of the Korean Physical Therapy Association [2]. The 5 most common subjects at the surveyed colleges were measurement and evaluation, physical agency therapy, physical therapy for the musculoskeletal system, physical therapy for the neurological system (including physical therapy for pediatric patients), and physical therapy for the cardiopulmonary system. Additionally, 2 future-oriented subjects, physical therapy for the integumentary system and community based physical therapy, were added to these major subjects. Each of the subject areas was designated by referring to the National Health Personnel Licensing Examination Board of Korea’s middle category titles for practical items [1] (Table 2).

## Establishment of learning objectives in a standard clinical practice curriculum

Based on the 2012 physical therapist job descriptions [3], an analysis of the clinical practice subjects related to the job of a physical therapist was conducted. The learning objectives for each subject were established by referring to a previous study [4] (Table 3).

Since 2014, the Korean physical therapist licensing examination has included 5 subjects covering 25 areas: basic physical therapy (anatomy and physiology, physical factor therapy, and public health), diagnosis and evaluation in physical therapy (principles of diagnostic evaluation, tests and evaluations, clinical decision-making, and physical therapy problem-solving), physical therapy interventions (musculoskeletal interventions, neurological interventions, cardiopulmonary system, integumentary system, and physical therapy problemsolving), medical law (medical law, medical technician law, welfare law for the disabled, welfare law for the elderly, and national health insurance law), and a practice examination (musculoskeletal system, neurological system, cardiopulmonary system, integumentary system, chronic and intractable diseases, community-based physical therapy, and measurement and evaluation) [1].

Clinical practice is useful for students to improve their interpersonal relationship skills with the members of a hospital organization and to cultivate the ability to cope with various situations. The training also helps students establish a specific career plan after graduation and develop the skills required for treating patients. Moreover, it enhances student empowerment, which contributes to more effective decision-making skills and self-efficacy [5].

In physical therapy education, clinical practice is an opportunity to apply theories learned in college in the clinical field and to learn how to apply specific skills. This is an important aspect of the curriculum, as it helps influence how the mindset of a professional physical therapist is established. It is critical for students to cultivate professional skills through clinical practice. Therefore, it is necessary to provide an environment with sufficient practical training to enhance practical skills and a professional mindset [6].

Through clinical practice, which is a crucial process in physical therapy education, students experience learning in various physical therapy fields, practice the basic roles and skills of a physical therapist, and acquire the ability to cope with the changing needs of patients [6]. In addition, previous research has proposed a standard curriculum for Korean colleges based on comparative analyses of overseas physical therapy curricula and clinical practice hours, with an emphasis on the necessity of increased clinical practice hours [7].

According to the study of Lee et al. [7] that analyzed the status of Korean physical therapy curricula, students fulfilled the average number of graduation credits specified by the Korea Physical Therapy Association. The study reported that no difference was found in the major curricula despite the fact that the curricula were dualized.

According to the report of Kim and Lee [6], the high-priority challenges that must be addressed to ensure effective clinical practice are as following, in descending order: difficulties in establishing excellent training institutions, the absence of university hospitals that meet the demands of the educational fields and common training programs, educational differences between schools and hospitals, and the absence of clinical practice quality control programs.

Practical programs presenting teaching methods (clinical practicums) between clinical instructors and students have been described in several advanced countries belonging to the World Confederation for Physical Therapy, such as USA, New Zealand, and Australia [7]. A standard curriculum for physical therapy clinical practice is necessary for the qualifying baseline of the licensing examination, so descriptions of acceptable examples of standard curricula among the advanced countries in World Confederation for Physical Therapy must be published, and suggestions should be made regarding the necessary period (hours) for clinical practice.

This study identified the subjects most commonly taught in colleges as standard subjects (musculoskeletal physical therapy, neurological physical therapy, cardiopulmonary physical therapy, and integumentary physical therapy).

The learning objectives of the standard practice curriculum were selected according to the basic job functions of a physical therapist. These functions included disease evaluation and intervention and training, as outlined in the 2012 physical therapist job descriptions.

## Limitations

The standard clinical practice curriculum was structured according to the subjective viewpoints of the researchers, based on preceding studies in the Republic of Korea and abroad and on a survey of domestic colleges. Therefore, these suggestions are limited to physical therapy education in the Republic of Korea.

In conclusion, we suggest a standard clinical practice curriculum and learning objectives for physical therapy education in Korea, as presented in Tables 2 and 3. It is also suggested that decisions related to the assignment of case-based items by major field, disease, and other classifications for the Korea Physical Therapy Licensing Examination be made as soon as possible.

## Figures and Tables

**Table 1.** Duration, subjects, credits, periods, and places of clinical practice in physical therapy education programs, collected from 14 universities and 19 colleges in Korea from June to September 2016

Contents		College	University
Duration, subjects, credits	Average duration (wk)	9.5	15
	Average subjects (numbers)	2.0	4.6
	Average credits (score)	8.4	12.4
Period	1st clinical practice	5	3
	1st, 2nd clinical practice	11	8
	1st, 2nd, 3rd clinical practice	3	3
	2nd year, winter vacation	13	
	2nd year, 2nd semester	1	
	3rd year, summer vacation	11	3
	3rd year, winter vacation		5
	3rd year, 1st semester	6	
	3rd year, 2nd semester	2	3
	4th year, summer vacation		4
	4th year, 1st semester		12
Place	University hospital	19	14
	General hospital	19	13
	Hospital	17	7
	Clinic	5	2
	Welfare center	3	2
	Public health center	2	
	Nursing hospital	13	4
	Children’s center	1	1
	Fitness center	1	2
	Others	4	3

**Table 2.** Status of the standard clinical practice curriculum of physical therapy education, based on a survey of 14 universities and 19 colleges in Korea from June to September 2016

Period (wk)	Subject	Category in the Korean physical therapist licensing examination	Present implementation at 33 universities/colleges (%)
1–2	Measurement & evaluation	Muscle testing	81.8
		Range of movement measurements	
3–4	Physical agents in rehabilitation	Pain relief	18.2
		Neuromuscular stimulation	
		Diathermy	
		Ultraviolet/infrared	
5–8	Physical therapy for the musculoskeletal system	Soft tissue injuries	77.8
		Skeletal tissue injuries	
		Diseases in the lumbar area	
		Diseases in the vasculature and nerves	
9–12	Physical therapy for the neurological system	Brain injuries and diseases	77.9
		Spinal cord injuries and diseases	
		Peripheral nerve injuries	
		Developmental movement disorders	
13–14	Physical therapy for the cardiopulmonary system	Diseases of the cardiac system	47.8
		Diseases of the pulmonary system	
15	Physical therapy for the integumentary system	Burns	19.2
		Lymph	
16	Community-based physical therapy (11%)	Patient education	11.4
		Environmental evaluation	

**Table 3.** Learning objectives related to the tasks in job descriptions (units: importance score; maximum of 5 points)

	Measurement and evaluation, physical agent	PT for equipment	Musculoskeletal PT	Neurological PT	Cardiopulmonary PT	Integumentary PT
Collecting data	4.18	4.18	4.18	4.18	4.18	
Medical history	4.18	4.18	4.18	4.18	4.18	
Muscle testing	4.17		4.17	4.17		
Examination of range of motion	4.27		4.27	4.27		
Examination of walking			4.25	4.25		
Examination of MS			4.03			
Examination of NS				3.93		
Examination of cardiopulmonary system					3.65	
Examination of integument system						3.68
PT using equipment		3.92	3.92	3.92	3.92	
Radio agent therapy	3.95					3.95
Hydro agent therapy	3.95					
PT for MS			4.04			
PT for NS				4.07		
Therapeutic exercises			4.04	4.04	4.04	4.04
Electro agent therapy	3.95		3.95	3.95		3.95
Adaptive training		3.92	3.92	3.92	3.92	3.92
PT for the cardiovascular system					4.04	
PT for the pulmonary system					4.04	
PT for the integument						3.95

PT, physical therapy; MS, musculoskeletal system; NS, neurological system.

## Supplementary material

Supplement 1. Data file is available from https://doi.org/10.7910/DVN/L3UIAJ.
